# Web-Based Public Reporting as a Decision-Making Tool for Consumers of Long-Term Care in the United States and the United Kingdom: Systematic Analysis of Report Cards

**DOI:** 10.2196/44382

**Published:** 2023-12-14

**Authors:** Kristina Kast, Sara-Marie Otten, Jens Konopik, Claudia B Maier

**Affiliations:** 1 Chair of Health Care Management Institute of Management Friedrich-Alexander-Universität Erlangen-Nürnberg Nürnberg Germany; 2 School of Public Health Universität Bielefeld Bielefeld Germany

**Keywords:** long-term care, medical decision-making, nursing homes, public reporting, quality improvement, report cards

## Abstract

**Background:**

Report cards can help consumers make an informed decision when searching for a long-term care facility.

**Objective:**

This study aims to examine the current state of web-based public reporting on long-term care facilities in the United States and the United Kingdom.

**Methods:**

We conducted an internet search for report cards, which allowed for a nationwide search for long-term care facilities and provided freely accessible quality information. On the included report cards, we drew a sample of 1320 facility profiles by searching for long-term care facilities in 4 US and 2 UK cities. Based on those profiles, we analyzed the information provided by the included report cards descriptively.

**Results:**

We found 40 report cards (26 in the United States and 14 in the United Kingdom). In total, 11 of them did not state the source of information. Additionally, 7 report cards had an advanced search field, 24 provided simplification tools, and only 3 had a comparison function. Structural quality information was always provided, followed by consumer feedback on 27 websites, process quality on 15 websites, prices on 12 websites, and outcome quality on 8 websites. Inspection results were always displayed as composite measures.

**Conclusions:**

Apparently, the identified report cards have deficits. To make them more helpful for users and to bring public reporting a bit closer to its goal of improving the quality of health care services, both countries are advised to concentrate on optimizing the existing report cards. Those should become more transparent and improve the reporting of prices and consumer feedback. Advanced search, simplification tools, and comparison functions should be integrated more widely.

## Introduction

An increasing number of people worldwide have functional or cognitive impairments and are dependent on others to manage their everyday lives [1]. This often requires one to move to a long-term care facility. However, the search for a suitable facility is often complex and calls for an assessment of information about the quality of care. Report cards with freely available information on facilities’ performance can help to make an informed decision.

The method of making health care quality information publicly available and transparent to consumers is called public reporting [2]. The aim is to improve the quality of health care services [3]. Public reporting is expected to work in 2 ways to achieve this goal. First, it shows providers how they perform compared with other providers and gives them the opportunity to undertake measures for performance improvements (change pathway). Second, public reporting enables consumers to compare facilities and distinguish well-performing from poor-performing providers, so they can choose the best one (selection pathway) [4-6]. As a consequence, this not only enables consumers to make an informed decision but also activates providers (motivation) to actually undertake improvement measures [4].

From an international perspective, the United States and the United Kingdom are 2 countries with a long tradition of public reporting [7]. In the United States, public reporting was initiated in the hospital sector in 1754 [8]. In 1984, the Institute of Medicine (today the National Academy of Medicine) drew attention to the poor quality of nursing homes [9], and public reporting was subsequently adopted within the long-term care sector. Since 1998, it has become mandatory for long-term care facilities to regularly submit selected facility and resident data to a national database (Minimum Data Set), on which quality indicators (eg, ulcer prevalence, restraint use, and mobility improvement) are analyzed by the Centers for Medicaid and Medicare (CMS) [9,10]. Since 2002, this information, which also includes inspection results performed by the CMS, has been published on the national report card Nursing Home Compare (Medicare.gov) [10,11].

In the United Kingdom, the reporting of hospital performance information dates back to 1860, when the first systematic reporting of hospital mortality rates began [8]. In the UK long-term care sector today, facilities are encouraged to monitor and report their own performance data to make quality transparent and to improve the quality of their services. It is mandatory for facilities to undergo the annual quality inspections performed by authorized health care agencies. In England, the Care Quality Commission (CQC), founded in 2008, is the agency regulating and inspecting health and social care [12]. The CQC assesses the facilities in terms of patient safety, effectiveness, and other aspects [13]. The results of these inspections are published on the CQC website [14] and on the report card NHS Choices [15]. In Scotland, the Care Inspectorate, established in 2011, is responsible for the registration and inspection of long-term care facilities [12,16]. It assesses the quality according to 6 grades (from “unsatisfactory” to “excellent”), considering aspects such as staffing or management quality [17,18]. The inspection results are available on the Care Inspectorate website [19].

By now, many new report cards have been created, which provide information on long-term care facilities [2]. By analyzing those report cards, this study can contribute to the body of literature by revealing the current situation of web-based public reporting as a decision-making tool for consumers and indicating further development potential for the countries themselves. It can also serve as a basis for a wide variety of further studies that assess “selection pathway”–measures in long-term care settings. As for the US- and UK-inspired report cards in other countries, it can indicate to what extent they should be reevaluated regarding their own public reporting.

Regarding academic discussions on public reporting, several international studies focused on long-term care and included the United States, the United Kingdom, or both in their comparisons. Some of them studied public reporting in general. For example, they compared the effectiveness [20] or validity [21] of quality indicators used in the long-term care sector of those countries, studied what quality information people prefer when choosing a long-term care facility [22], and gave implications for more effective public reporting [23].

There are also some international studies with a focus on web-based public reporting. For example, du Moulin and colleagues [24] compared the official websites of the quality initiatives of 14 countries and found that the quality indicators on them varied in type and number across the countries, while the outcome indicators received little attention. Similarly, Rodrigues and colleagues [3] provided a comparative overview of quality indicators published on selected websites of 7 countries and appealed for better design of report cards. Rechel and colleagues [25] surveyed key national informants of 11 countries about quality information the countries published on report cards. The study had, however, a strong focus on hospitals. On long-term care, the authors reported that only a few countries displayed quality information as an overall rating. Damman and colleagues [26] conducted an internet search on 10 countries and found 42 report cards. The authors reported that many of them offered no quality information at all. This study, however, also covered different health care sectors.

While there are several international comparative studies on long-term care public reporting in different countries, including the United States and the United Kingdom, they either did not focus on report cards [20-23], addressed only selected report cards [3,24], or combined report cards of different health care sectors [25,26]. With this study, we aimed to analyze the current state of public reporting in the United States and the United Kingdom, with a focus on report cards for the search of long-term care facilities. Therefore, we addressed the following questions: (1) What report cards are available for consumers in the United States and the United Kingdom when using the internet to choose a long-term care facility? and (2) What types of quality information do these report cards provide and how?

## Methods

### Search Strategy

We decided on the United States and the United Kingdom due to their long experience with public reporting but also because of language understanding. The fundamentals of similarly designed studies from other settings and countries served as the structural basis of this study. We systematically searched for the US and UK websites, which provide information on the performance of long-term care facilities to the public. The search was conducted in mid-2020 and updated in mid-2021. As research suggests, we identified the 2 most popular search engines [27]. Google and Bing had the highest market shares based on page views referred by a search engine in both countries at that time [28]. To avoid bias in accessing the search engines due to the location of the researchers in Germany, we used internet access through the virtual private network CyberGhost [29], to simulate the search from the United States and the United Kingdom. In addition, to avoid potential influence from past search behavior, we set up a new version of a browser for the search. Before drawing a sample of websites, we constructed a search strategy following previous research [27]. We used terms such as “compare nursing homes,” “nursing home rating,” “find care homes,” and “choose the right care home” (see Table S1 in Multimedia Appendix 1 for more details), following previous literature [30-32].

### Sampling of Websites

Search engines typically display 10 hits per search query by default. The upper organic hits (ie, without ads) are usually the most noticed. After that, the likelihood of being noticed decreases [33]. Furthermore, over 70% of clicks are made on the first page, and from the third page onward, the click probability is less than 2% [34]. For these reasons, we considered only the first 50 hits per search engine, per search term, and per country, which resulted in a total sample of 1500 websites.

After removing duplicates, we excluded websites not related to health care. We then checked each individual link based on predefined inclusion criteria. We were interested in websites that allowed users to search for a facility nationwide, provided access to quality information without registration, and gave users the possibility to choose from a range of facilities from different providers (ie, not the home pages of single providers). Those websites had to provide at least 1 instance of quality information, which could be either objective (eg, pressure ulcer) or subjective (eg, consumer reviews) [35]. We excluded websites if they provided no quality information but only background information about long-term care or if they reported quality information only on other types of care (eg, respite care or home care).

### Sampling of Facility Profiles

As suggested by Kast and colleagues [31], on the report cards that met the inclusion criteria, we searched for long-term care facilities in specific cities. Due to the geographic size and population of the countries (the United States with over 300 million inhabitants and the United Kingdom with about 65 million) [36], 4 cities were chosen for the United States and 2 for the United Kingdom. For the former, we chose New York as a metropolitan city with millions of inhabitants and a high price segment. Miami, Portland, and Denver were chosen as medium-sized cities with between 500,000 and 1 million inhabitants. These cities are in the interior or on the coasts of the country. They have a very high proportion of internet users as well as a growing and similar proportion of older people to the national population [36,37]. Portland and Miami are among the most popular areas for retirement [38]. Similarly, in the United Kingdom, London as a metropolitan city covers the southern part, and Glasgow as a medium-sized city covers the northern part of the country. Compared to the national average [36], the 2 cities have a higher-than-average proportion of older people. Both cities have a large proportion of internet users [39].

For each included report card and city, we considered a sample of 10 facilities as suggested by Kast and colleagues [31]. As we intended to obtain a comprehensive view of the information report cards are able to provide, we needed to analyze more sophisticated profiles. We expected to find such profiles in the described areas rather than in rural regions. In cities, the competition between facilities might be more intense and consumers might be more engaged in using internet-based information. Both can lead to a more active use of profiles by facilities. In total, we screened 1320 facility profiles. Based on those profiles, we analyzed the information provided by the included report cards.

### Data Extraction and Analysis

**Figure 1:**
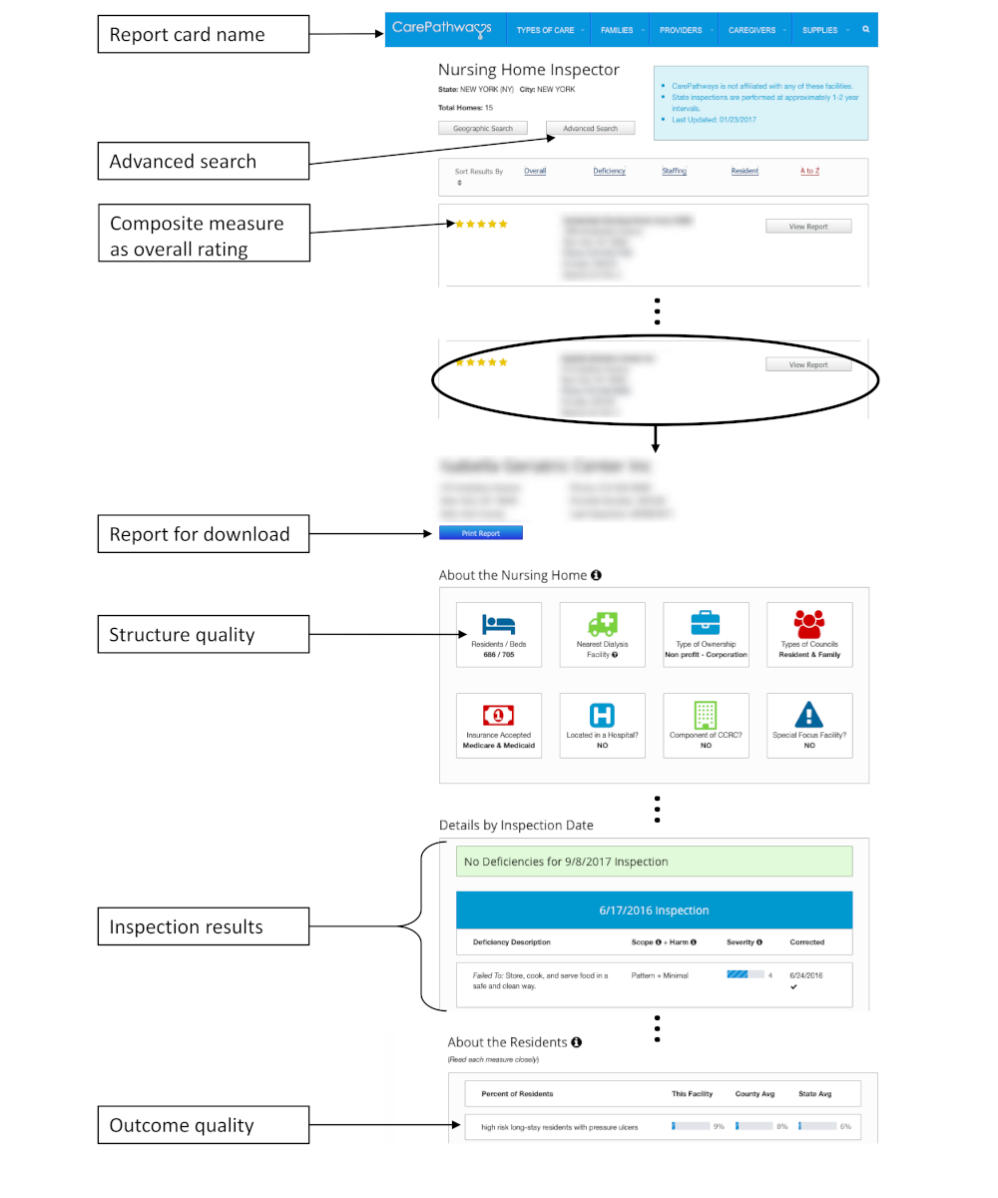
Example of report card elements (shortened for better comprehensibility).

Figure 1 shows an example of a report card and highlights some relevant elements of the data extraction on this report card. From each included report card, we first collected general information, for example, as suggested by Emmert and colleagues [35], information websites reported about themselves—the intention of the report card (focus) and the origin of the information (data source). We then collected information about the functional scope, as suggested by Kast and colleagues [31]. We checked whether users could communicate their satisfaction with a facility to others. In addition, we examined the possibilities of customizing the search. It included a simple search field or an advanced search. The former generally requires users to specify what they are looking for and where, for example, a nursing home under a specific postal code. An advanced search allows the users to specify the search in more detail with additional customization of distance, facility type, or both. Furthermore, we considered simplification tools, which allow users to handle the complexity of information [40]. Such tools could allow for adjusting quantity per page or sorting and filtering the hit list. Additionally, we checked for the ability to compare several facilities with each other.

We checked whether quality information was available. For objective quality, we extracted the following 3 types of information based on the work of Donabedian [41]: structure, process, and outcome quality. These types have been used in quality-of-care studies [26,42-44]. In this study, structural quality, for example, refers to location, wheelchair accessibility, staffing ratio, and facility equipment. Process quality covers aspects such as the use of physical restraint, pressure ulcer prophylaxis, or the involvement of relatives in decision-making processes. Outcome quality encompasses, for example, the number of residents with pressure ulcers or unintentional weight loss. As suggested by previous studies [31,35], we added further types to these 3 types of information. We checked the availability of price information on report cards. We also examined whether the inspection results based on the official quality checks (eg, from the CMS, CQC, or other quality initiatives) were presented. For subjective information, we examined whether consumer experience and satisfaction data (consumer feedback) were displayed.

On report cards with inspection results, we analyzed the presentation format of this information. We distinguished composite measures [35] (eg, a 5-star quality rating), either for different areas (eg, health outcomes of residents, staffing, or nursing care) or for the overall quality of a facility. We also checked for alternative information presentations, such as a downloadable quality report [31], or a link to another website (eg, official CMS website). On report cards, which provided consumer feedback, we distinguished between presentation formats such as a scaled quality assessment of defined areas (eg, food quality) and free-text comments, as suggested by Emmert and colleagues [35].

All mentioned data were extracted in Microsoft Excel (Microsoft Corporation) sheets using the described types of quality information, presentation formats, and functions. Any described type was considered covered as soon as we observed at least 1 instance of quality information, presentation format, or function associated with the respective type. Thus, for each type, we stated whether it was covered on the respective website (yes or no) [30]. After data extraction, each type was analyzed descriptively using total numbers and percentages.

### Ethical Considerations

Ethics approval was not required for this study since all data was publicly available and there were no human participants.

## Results

### Identified Report Cards

We identified 40 report cards (Table 1): 26 (65%) in the United States and 14 (35%) in the United Kingdom (for URL and provider information of the report cards see Table S2 in Multimedia Appendix 1). In total, no report card restricted the search to long-term care facilities only (Table 2). Instead, the websites also included other care services for older people (eg, hospice care or home care), other providers (eg, physicians or hospitals), or nonmedical services (eg, hairdressers). In the United States, most (n=17, 65%) report cards focused on care services for older people, compared with less than half (n=6, 43%) in the United Kingdom.

**Table 1:** Process and result of report card identification.

Phases and processes		US report cards, n	UK report cards, n
**Identification**			
	Drawn sample	600	900
**Removal**			
	Duplicates	313	445
Remaining after removing duplicates		287	455
**Screening by selection criteria**			
	Not health care	28	135
	Background information	146	271
	Not nationwide search	56	11
	Registration required	6	4
	Error	25	15
	Hand search	6^a^	6^a^
Potentially relevant after screening		32	25
**Data collection**			
	Background information	1	2
	Not nationwide search	1	8
	Other kinds of facility	4	0
	Not available anymore	0	1
Report cards included		26	14

^a^Identified during the screening phase and added for the data collection phase.

**Table 2:** General information about report cards.

Information			US report cards (N=26), n (%)		UK report cards (N=14), n (%)		Total report cards (N=40), n (%)
**Focus on**							
	Providers of care services for older people	17 (65)		6 (43)		23 (58)	
	Different health care providers	2 (8)		2 (14)		4 (10)	
	No specific focus on health care	7 (27)		6 (43)		13 (33)	
**Data source**							
	Inspections results (eg, Centers for Medicaid and Medicare)	6 (23)		2 (14)		8 (20)	
	Consumer or provider information (eg, comments or provider registration data)	10 (38)		4 (29)		14 (35)	
	Inspection results combined with consumer or provider information	4 (15)		1 (7)		5 (13)	
	Copied from publicly available information (eg, Nursing Home Compare website)	2 (8)		N/A^a^		2 (5)	
	Not reported	4 (15)		7 (50)		11 (28)	

^a^N/A: Not applicable.

In both countries, the data provided on the report cards had different origins (Table 2 and Table S3 in Multimedia Appendix 1). Most of the report cards (n=10, 38% in the United States and n=4, 29% in the United Kingdom) indicated that they use data provided by service consumers (eg, user comments) and service providers (eg, nursing home providers registered on the respective website) only. Another often-reported data source was the data from inspections conducted by government agencies (n=6, 23% in the United States and n=2, 14% in the United Kingdom). Some report cards combined both types of data sources (n=4, 15% in the United States and n=1, 7% in the United Kingdom). On half of the UK websites (n=7, 50%), no information on the data source was given. On the US websites, this was the case for only 4 (15%) websites.

### Functions of Report Cards

Functions are shown in Table 3 and Table S4 in Multimedia Appendix 1. While a simple search function was available on all identified report cards, only one-fifth (n=7, 18%) of them in both countries (n=2, 14% of those in the United Kingdom) offered an advanced search field. Other functions were equally available in both countries. In total, on more than half of the websites (n=24, 60%), users could customize the hit list using various simplification functions after the search has been performed. The sorting option was the most frequent (n=21, 88%) simplification function, followed by filters (n=15, 63%), and the possibility to change the number of facilities shown (n=3, 13%). An even rarer (n=3, 8%) function was the ability to compare multiple potential facilities on the internet. In contrast, the option for users to leave feedback on a particular facility was offered by about half of the report cards (n=21, 53%). After registration, users could rate facilities according to certain predefined criteria (eg, friendliness of staff and quality of food) or leave a free-text comment.

**Table 3:** Functions on websites.

Function				US report cards (N=26), n/N (%)		UK report cards (N=14), n/N (%)		Total report cards (N=40), n/N (%)
**Kind of search field**								
	Single		26/26 (100)		14/14 (100)		40/40 (100)	
	Advanced		5/26 (19)		2/14 (14)		7/40 (18)	
**Simplification tools**								
	No		11/26 (42)		5/14 (36)		16/40 (40)	
	**Yes**		15/26 (58)		9/14 (64)		24/40 (60)	
		Sort	13/15 (87)		8/9 (89)		21/24 (88)	
		Filter	8/15 (53)		7/9 (78)		15/24 (63)	
		Adjust	2/15 (13)		1/9 (11)		3/24 (13)	
**Internet-based comparison**								
	No		24/26 (92)		13/14 (93)		37/40 (92)	
	Yes		2/26 (8)		1/14 (7)		3/40 (8)	
**Consumer feedback allowed**								
	No		12/26 (46)		7/14 (50)		19/40 (47)	
	Yes		14/26 (54)		7/14 (50)		21/40 (53)	

### Quality Information and Its Presentation

Table 4 and Table S5 in Multimedia Appendix 1 show details on types of quality information and presentation formats. All report cards showed information on structural quality. In total, less than half of them (n=15, 38%) reported information about processes in facilities; this kind of information was found more frequently in the United Kingdom (n=7, 50%) than in the United States (n=8, 31%). On the contrary, information on the quality of outcomes was only reported in the United States (n=8, 31%). Prices were reported on one-third (n=12, 30%) of all report cards. The subjective information “consumer feedback,” on the other hand, was (with the exception of the structural quality) in both countries more common (n=27, 68%) than objective quality information.

**Table 4:** Types of quality information and presentation format.

Quality information and presentation format				US report cards (N=26), n/N (%)		UK report cards (N=14), n/N (%)		Total (N=40), n/N (%)
**Structure**								
	No		0/26 (0)		0/14 (0)		0/40 (0)	
	Yes		26/26 (100)		14/14 (100)		40/40 (100)	
**Process**								
	No		18/26 (69)		7/14 (50)		25/40 (62)	
	Yes		8/26 (31)		7/14 (50)		15/40 (38)	
**Outcome**								
	No		18/26 (69)		14/14 (100)		32/40 (80)	
	Yes		8/26 (31)		0/14 (0)		8/40 (20)	
**Prices**								
	No		19/26 (73)		9/14 (64)		28/40 (70)	
	Yes		7/26 (27)		5/14 (36)		12/40 (30)	
**Inspection results**								
	No		10/26 (38)		6/14 (43)		16/40 (40)	
	**Yes**		16/26 (62)		8/14 (57)		24/40 (60)	
		Overall^a^	15/16 (94)		5/8 (63)		20/24 (83)	
		By area^b^	14/16 (88)		6/8 (75)		20/24 (83)	
		Details^c^	8/16 (50)		1/8 (13)		9/24 (38)	
		Report^d^	3/16 (19)		0/8 (0)		3/24 (13)	
		Link^e^	1/16 (6)		7/8 (88)		8/24 (33)	
**Consumer feedback**								
	No		8/26 (31)		5/14 (36)		13/40 (32)	
	**Yes**		18/26 (69)		9/14 (64)		27/40 (68)	
		Scaled	18/18 (100)		9/9 (100)		27/27 (100)	
		Comment	13/18 (72)		7/9 (78)		20/27 (74)	

^a^Composite measure as overall rating.

^b^Composite measure as rating by area (eg, health inspections, staffing, and resident care).

^c^Detailed information from inspections by area.

^d^Reports for download.

^e^Link to the authority website with detailed inspection result.

On all 27 report cards with consumer feedback, it was presented in the form of scaled ratings. This means that certain predefined areas, such as cleanliness or food quality, were rated by users on a scale and displayed in the form of stars, for example. Many (n=20, 74%) of those ratings were supplemented by comments written by users in free text. The quality of structure, processes, and outcomes is usually information from inspection controls. Far more than half (n=24, 60%) of the identified report cards made use of this information. Most of them were presented as composite measures, which showed the overall quality (n=20, 83%) or were separated by area (n=20, 83%) such as health outcomes, staffing, or nursing care. In the United Kingdom, most of the 7 report cards with inspection controls–based information displayed it as a link to the website of the responsible authority (eg, the CQC or Care Inspectorate), where users could see inspection results in more detail. The US websites provided such links less often (n=1, 6%), but in some cases, they provided such information in detail on the respective report card (n=8, 50%) or as a complete CMS report with inspection results as a file for download (n=3, 19%).

## Discussion

### Principal Findings

Many people need to move to a long-term care facility. Web-based public reporting can help find a suitable one. With this study, we aimed to analyze the current state of such report cards in the United States and the United Kingdom, 2 countries with a long tradition of public reporting. We found 40 report cards, which allowed a nationwide search for a long-term care facility and provided free access to quality information. This study shows that many aspects were well covered on those websites. Most report cards focused explicitly on long-term care, structural quality information was always provided, users had the possibility to share experiences, and medical information from inspections was always displayed as composite measures. However, our findings suggest that there are also some deficits on many report cards in the United States and the United Kingdom. Both countries poorly communicated the source of data and sparingly reported prices and consumer feedback. Many of them did not provide an advanced search function, simplification tools, or comparison functions.

Kumpunen and colleagues [2] reported several years ago about the rapid and substantial growth of the number of websites. This required an extra website to guide users to find the relevant information. Considering this, we expected to find a greater number of report cards. However, we also know that consumers often use only 1 report card as a basis for decision-making [45]. Therefore, it might be even more important to optimize existing report cards instead of expanding their number. Below, we explain the problems associated with the stated deficits and provide important implications for improvement.

### Implications

As mentioned above, the aim of public reporting is to improve the quality of health care services [5]. Providers are those who can undertake measures to change their services [6]. However, as emphasized by Berwick and colleagues [4], this strongly depends on the intrinsic motivation to perform better than others and is not a reliable strategy in a complex system such as health care. Conclusively, the “selection pathway” is even more important as it creates pressure through the consequences of the choice by consumers and motivates providers to undertake improvement measures [4]. However, the realization of this pathway can fail because users are dissatisfied with report cards and do not take them into account when making the final decisions [46]. For this reason, it is necessary to make report cards more helpful for users [47,48].

First, it is important to be transparent about where the data came from since this is an essential aspect of credibility [49-51]. We found that in total, 11 (28%) of the 40 report cards, and especially many (7/14, 50%) in the United Kingdom, did not state the source of the provided information. Not reporting the data source can represent a great obstacle to the effectiveness of public reporting instruments [52], as consumers doubt the trustworthiness of the provided quality information and often refrain from using report cards [50]. Thus, we recommend that both countries and especially the United Kingdom, more clearly communicate the source of data they use.

Second, some types of information are more important to users than others. Research shows that consumers are more likely to decide on such criteria as prices or recommendations than on medical information [53-56]. Thus, to be useful for consumers, report cards should provide quality information that is of interest to them. In both countries, prices in this sector are a relevant criterion when choosing a facility, as the monthly out-of-pocket payments are high (US $7908 in the United States [57] and £3552 [US $4280] in the United Kingdom [58]). We found, however, that websites insufficiently reported prices (12/40, 30%). Particularly in the United States, there is room for improvement in price transparency, considering that only 7 (27%) of 26 report cards showed price information, and 3 (43%) of those 7 would only reveal details after registration. Similarly, consumer feedback was missing on one-third (13/40, 32%) of the identified report cards in this study. Hefele and colleagues [59] emphasize that the lack of subjective perspective on report cards pushes consumers to use information on social media, yet its validity is still unclear [60,61]. Consumer feedback can be a valuable supplement to objective quality information; it can strengthen the attractiveness of public reporting and improve the quality of decision-making [62-64]. Thus, especially in the United States, price transparency should be improved. In both countries, more report cards should supplement the objective quality information with subjective consumer feedback.

Third, it is not enough to show the content users prefer to help them make an informed decision. Sometimes the amount of information can be overwhelming for users [65]. Different studies showed that people wish for a lot of different quality information, but it increases the complexity without necessarily improving decision-making [65-70]. To counteract this problem of overestimating one’s own ability to process large amounts of information, it is important for report cards to include customizable formats [40]. In this study, however, only a few report cards allowed for customization. At the beginning of the search process, most report cards only offered a simple search function. While this function may have different levels of technical sophistication and deliver different results depending on search behavior [71], an advanced search would give users more possibilities to customize the search based on their own preferences and would help them find relevant information more easily and more quickly. To reduce the complexity during the screening of the hit list, simplification tools could be helpful [40]. This study shows that many report cards used at least 1 of such tools (eg, filters), but 40% (16/40) did not.

Furthermore, users may find it difficult to compare multiple potential facilities from a hit list. An internet-based comparison of several facilities allows users to get a first impression of the performance of the facility in question and to avoid errors in the interpretation of the quality information [72]. In addition, users do not have to gather information themselves in a time-consuming manner [73]. Although the comparison function on report cards seems to be a useful function according to the literature, this study shows that this function was especially rare on report cards in both countries (2 in the United States and 1 in the United Kingdom). Thus, we recommend that website managers in both countries more intensively integrate the advanced search function and simplification tools on the websites and more often provide the opportunity to compare several potential facilities.

All in all, it seems that, for several reasons, web-based public reporting in its current form cannot be a helpful decision-making tool for consumers. At present, users do not have the opportunity to assess the trustworthiness of information, the displayed quality information does not always correspond to their preferences, and users are not provided with enough functions to handle the complexity on report cards. Improving the identified websites would not only help consumers make an informed decision on the selection of a long-term care facility (selection pathway [4]). Moreover, it could make the other parts of the chain suggested by Berwick and colleagues [4] work. By the consequences of the choice, it could increase the odds of activating the motivation of providers to act on quality improvements, and thus, to improve the quality of health care services.

### Significance of the Study

This study reveals that addressing the current deficiencies can contribute to more effective public reporting and improved long-term care services for older people who are dependent on others. According to our assessment, many of these specific problems are easy to fix (eg, functions). The study benefits various groups associated with long-term care. As for the practical side, this study points report card managers to the most pressing issues, helps health care agencies better understand the information preferences of consumers, and motivates long-term care facilities to provide more quality information on their profiles on report cards. From a theoretical standpoint, our findings provide a possible explanation for why report cards are still poorly used by consumers. Further studies could work on the optimization of report cards by testing different formats using inferential statistics. Our findings can serve as a basis for such studies. Based on experiences from the United States and the United Kingdom, other countries (eg, Germany) adopted public reporting in institutional long-term care. This implies that similar strengths and weaknesses of web-based public reporting could be identified in those countries, which limits its ability to serve as a helpful decision-making tool for consumers. We encourage other countries to evaluate their current state of public reporting. This paper provides an extensive blueprint for such a reflection by highlighting elements of different areas of web-based public reporting that should be addressed.

### Limitations

When interpreting and using the findings from this study, some aspects should be kept in mind. Although we updated our data last year, websites are subject to short-term changes at any time. Therefore, it is possible that the availability of websites and the status of functions and information on them today differ from those at the time of the data collection. In this study, we defined outcome quality as concrete objective indicators (eg, number of falls). This has resulted in a lack of outcome quality being identified in the United Kingdom. However, to avoid misinterpretation, it should be kept in mind that the United Kingdom addresses these types of facts in a different way (eg, safety). Furthermore, since it was not the subject of this study, we did not examine to what extent a type of quality information (eg, structural quality) was available on individual websites but only whether it was encountered at least once. We also did not evaluate in which format the quality information within a category was displayed (eg, numbers, graphs, or words). Further research could investigate these problems to give more specific recommendations for improvement for the managers of the identified report cards. In addition, we only included websites that allowed a nationwide search. Both countries, however, also have many report cards that are state-specific (eg, Aging and Disability Resource Connection of Oregon [74] and CQC [14]). As we assume that consumers should have the possibility to choose from a range of existing facilities, we decided to define our selection criteria this way. Thus, our findings are not generalizable to the whole field of web-based public reporting in long-term care, but only to those covering a nationwide search. Finally, we selected the United States and the United Kingdom due to their long tradition of public reporting and some decades of research on this topic, which is a strength of our study. The Netherlands, however, also has a lot of experience with public reporting [75] and would complement our research. Due to the risk of misinterpreted translations, we decided to focus on a smaller set of countries.

### Conclusions

All in all, it seems that web-based public reporting in its current form cannot be a helpful decision-making tool for consumers. At present, users do not have the opportunity to assess the trustworthiness of information, the displayed quality information does not always correspond to their preferences, and users are not provided with enough functions to handle the complexity on report cards. Both countries, but especially the United Kingdom, should become more transparent about the source of data they use; especially in the United States, price transparency should improve. In both countries, more report cards should supplement the objective quality information with subjective consumer feedback. Report card managers in both countries should more intensively integrate the advanced search function and simplification tools on the websites and more often provide the opportunity to compare several potential facilities. These improvements could not only make the report cards a more helpful decision-making tool for users but also bring public reporting a bit closer to its goal of improving the quality of health care services.

## Appendix

Multimedia Appendix 1 Additional information on methodology and results.

## Abbreviations

CMS: Centers for Medicaid and Medicare
CQC: Care Quality Commission
