# Supplementary material for: Web-Based Public Reporting as a Decision-Making Tool for Consumers of Long-Term Care in the United States and the United Kingdom: Systematic Analysis of Report Cards
Source: JMIR Form Res. 2023 Dec 14;7:e44382. doi: 10.2196/44382 (PMC10755662; doi:10.2196/44382)
Supplement: Multimedia Appendix 1 [file formative_v7i1e44382_app1.pdf]

Web-Based Public Reporting as a Decision-Making Tool for Consumers of Long-Term Care in the United States and the United Kingdom: Systematic Analysis of Report Cards

This is a Multimedia Appendix to a full manuscript published in the JMIR Formative Research

For citation information see http://doi.org/10.2196/44382

Table S1. List of search terms.

Search terms	
**USA**	**UK**
compare nursing homes	compare care homes
search for nursing home	find care home
find nursing home	choose the right care home
nursing home rating	care home rating
best nursing home	best care home
choose nursing home	compare residential homes
	find residential home
	best residential home
	choose residential home

Table S2. Identified report cards.

	USA	UK
	**Name of the report card**	**Name of the report card**
	(Provider)	*(Provider)*
**Nr.**	URL	URL
1	**Assisted Senior Living**	**Autumna**
	(AssistedSeniorLiving)	*(Autumna Ltd)*
	assistedseniorliving.net	autumna.co.uk
2	**Bing**	**BestCare Compare**
	(Microsoft Corporation)	*(iCaps Health Ltd)*
	bing.com	bestcarecompare.com
3	**Caregiverlist**	**Bettercaring**
	(Caregiverlist, Inc)	*(Valuing Care Financial Management)*
	Caregiverlist.com	bettercaring.com
4	**CarePathways**	**Bing**
	(Clare Absher and Casey Forbes)	*(Microsoft Corparation)*
	carepathways.com/nursing-homes	bing.com
5	**CareWindow**	**Carehome.co.uk**
	(NR)	*(Tomorrow's Guides Ltd)*
	carewindow.com	carehome.co.uk
6	**Caring.com**	**Caresourcer**
	(Caring LLC)	*(Caresourcer Ltd)*
	caring.com	caresourcer.com
7	**CiteHealth**	**Google**
	(Open Data Systems, Inc)	*(Google LLC)*
	citeheath.com	google.co.uk
8	**FamilyAssets**	**Hotfrog**
	(FamilyAssets Group LLC)	*(Moboom Ltd.)*

	familyassets.com	hotfrog.co.uk
9	**Google**	**Housingcare.org**
	(Google LLC)	*(Elderly Accommodation Counsel's)*
	google.com	housingcare.org/index.aspx
10	**LocalNursingHomes**	**MostRecommended Care**
	(The Local Network LLC)	*(Cade Directories Limited)*
	local-nursing-homes.com	mostrecommendedcare.co.uk
11	**MatchNursingHomes.org**	**Trustedcare**
	(MatchNursingHomes.org)	*(Search Care Limited trading)*
	matchnursinghomes.org	trustedcare.co.uk
12	**MedicaidPlanningAssistance.org**	**Which?**
	(ElderCare Resource Planning, LLC)	*(Which? Ltd)*
	medicaidplanningassistance.org/find-medicaid-nursing-homes	which.co.uk
13	**Nursing Home Compare**	**Yahoo**
	(U.S. Centers for Medicare & Medicaid Services)	*(Yahoo! Inc.)*
	medicare.gov/nursinghomecompare/search.html	uk.search.yahoo.com
14	**Nursing Home Inspect**	**Yell**
	(Pro Publica Inc.)	*(Yell ltd)*
	projects.propublica.org/nursing-homes	yell.com
15	**Nursinghomerating**	
	(nursinghomerating.org)	-
	nursinghomerating.org	
16	**NURSINGHOMEREPORT.org**	
	(United States Department of Health)	-
	nursinghomereport.org	
17	**Openplacement (ENSOCARE CHOICE)**	
	(Ensocare Inc.)	-
	openplacement.com	
18	**SeniorAdvice**	
	(Zero Four Media, LLC)	-
	senioradvice.com	
19	**SeniorAdvisor.com**	
	(A Place for Mom, Inc.)	-
	senioradvisor.com	
20	**SeniorCare.com**[#]	
	(SeniorCare.com)	-
	seniorcare.com	
21	**SeniorHomes.com**	
	(SeniorHomes.com, LLC)	-
	seniorhomes.com	
22	**The Nursing Home Site**	
	(NR)	-
	nursinghomesite.com	
23	**U.S.News**	
	(Inform U.S. News & World Report, L.P.ation and Advisory services provided by Caring.com)	-
	health.usnews.com/best-nursing-homes	
24	**Yahoo!**	
	(Verizon Media)	-

	search.yahoo.com	
25	**Yelp**	
	(Yelp Inc.)	-
	yelp.com	
26	**YP**	
	(Thryv Inc.)	-
	yellowpages.com	

Old domain: skillednursingfacilities.org
NR: Not reported

Table S3. General information about report cards.

		Focus on	Data source
**Name of the report card in the United States (n=26)**			
	Assisted Senior Living	a	d
	Bing	c	e
	Caregiverlist	a	f
	CarePathways	a	NR
	CareWindow	a	e
	Caring.com	a	e
	CiteHealth	b	e
	FamilyAssets	a	f
	Google	c	e
	LocalNursingHomes	a	g
	MatchNursingHomes.org	a	g
	MedicaidPlanningAssistance.org	a	NR
	Nursing Home Compare	b	d
	Nursing Home Inspect	c	d
	Nursinghomerating	a	d
	NURSINGHOMEREPORT.org	a	d
	Openplacement (ENSOCARE CHOICE)	a	NR
	SeniorAdvice	a	e
	SeniorAdvisor.com	a	e
	SeniorCare.com	a	d
	SeniorHomes.com	a	NR
	The Nursing Home Site	a	f
	U.S.News	c	f
	Yahoo!	c	e
	Yelp	c	e

	YP	c	e
**Name of the report card in the United Kingdom (n=14)**			
	Autumna	a	NR
	BestCare Compare	b	NR
	Bettercaring	a	f
	Bing	c	e
	Carehome.co.uk	a	NR
	Caresourcer	b	NR
	Google	c	e
	Hotfrog	c	NR
	Housingcare.org	a	NR
	MostRecommended Care	a	e
	Trustedcare	a	d
	Which?	c	d
	Yahoo	c	NR
	Yell	c	e

"a" represents providers of care services for older people. "b" represents different health care providers. "c" represents no specific focus on health care. "d" represents inspections results (eg, Centers for Medicaid and Medicare). "e" represents consumer/provider information (eg, comments and provider registration data). "f" represents inspections results combined with consumer/provider information. "g" represents copied from publicly available information (eg, Nursing Home Compare Website). NR: Not reported.

Table S4. Functions on websites.

Name of the report card		Kind of search field	Simplification tools	Internet-based comparison	Consumer feedback allowed
**In the United States (n=26)**					
	Assisted Senior Living	Single	Sort and adjust quantity per page	No	Yes[a]
	Bing	Single	No	No	No
	Caregiverlist	Single and advanced	Sort and filter	No	Yes[a]
	CarePathways	Single and advanced	Sort	No	No
	CareWindow	Single	Sort and filter	No	Yes[a]
	Caring.com	Single	No	No	Yes[a]
	CiteHealth	Single	No	No	Yes[a]

	FamilyAssets	Single and advanced	Sort	No	Yes[a]
	Google	Single	Filter	No	Yes[a]
	LocalNursingHomes	Single	Sort	No	Yes[a]
	MatchNursingHomes.org	Single	No	No	No
	MedicaidPlanningAssistance.org	Single	Sort and adjust quantity per page	No	No
	Nursing Home Compare	Single and advanced	Sort	Max. 3 facilities	No
	Nursing Home Inspect	Single and advanced	Sort and filter	No	No
	Nursinghomerating	Single	No	No	No
	NURSINGHOMEREPORT.org	Single	Sort	>20 facilities	No
	Openplacement (ENSOCARE CHOICE)	Single	No	No	No
	SeniorAdvice	Single	No	No	Yes[a]
	SeniorAdvisor.com	Single	Filter	No	Yes[a]
	SeniorCare.com	Single	No	No	No
	SeniorHomes.com	Single	No	No	Yes[a]
	The Nursing Home Site	Single	No	No	Yes[a]
	U.S.News	Single	Sort and filter	No	No
	Yahoo!	Single	No	No	No
	Yelp	Single	Sort and filter	No	Yes[a]
	YP	Single	Sort and filter	No	Yes[a]
**In the United Kingdom (n=14)**					
	Autumna	Single	Sort and filter	No	No
	BestCare Compare	Single	Sort and filter	No	Yes[a]
	Bettercaring	Single	Sort	No	No
	Bing	Single	No	No	No
	Carehome.co.uk	Single and advanced	Sort and filter	Max. 15 facilities	Yes[a]
	Caresourcer	Single	Sort and filter	No	No
	Google	Single	Filter	No	Yes[a]
	Hotfrog	Single	No	No	Yes[a]

	Housingcare.org	Single and advanced	Sort and adjust quantity per page	No	Yes[a]
	MostRecommended Care	Single	No	No	Yes[a]
	Trustedcare	Single	No	No	No
	Which?	Single	Sort and filter	No	No
	Yahoo	Single	No	No	No
	Yell	Single	Sort and filter	No	Yes[a]

[a]Personal data required.

Table S5. Types of quality information and presentation format.

		Structure	Process	Outcome	Prices	Inspection Results	Consumer feedback
**Name of the US report card (n=26)**							
	Assitedseniorliving	Yes	Yes	No	No	No	Scaled and comment
	Bing	Yes	No	No	No	No	Scaled
	Caregiverlist	Yes	No	No	Yes	Yes (1,2)	No
	Carepathways	Yes	No	Yes	No	Yes (1-4)	Scaled
	Carewindow	Yes	No	No	No	Yes (1,2)	Scaled and comment
	Caring.com	Yes	Yes	No	No	No	Scaled and comment
	Citehealth	Yes	Yes	Yes	No	Yes (1-3)	Scaled and comment
	Familyassets	Yes	Yes	No	Yes	No	Scaled and comment
	Google	Yes	No	No	No	No	Scaled and comment
	Localnursinghomes	Yes	No	Yes	No	Yes (1,2)	Scaled and comment
	Matchnursinghomes	Yes	No	Yes	No	Yes (1-3)	No
	Medicaidplanning assistance	Yes	No	No	No	Yes (1)	Scaled
	Nursing Home Compare	Yes	No	Yes	No	Yes (1-4)	No

	Nursinghomeinspect	Yes	No	No	No	Yes (5)	No
	Nursinghomerating	Yes	No	Yes	No	Yes (1-3)	No
	Nursinghomereport	Yes	No	No	No	Yes (1,2)	No
	Nursinghomesite	Yes	Yes	No	Yes	Yes (1-3)	Scaled
	Openplacement	Yes	No	No	No	Yes (1,2)	No
	Senioradvice	Yes	Yes	No	Yes	Yes (1,2)	Scaled and comment
	Senioradvisor	Yes	Yes	No	Yes	No	Scaled and comment
	Seniorcare	Yes	No	Yes	No	Yes (1-3,5)	Scaled and comment
	Seniorhomes	Yes	Yes	No	Yes	No	Scaled and comment
	US-News	Yes	No	Yes	No	Yes (1-4)	No
	Yahoo	Yes	No	No	No	No	Scaled
	Yelp	Yes	No	No	Yes	No	Scaled and comment
	YP	Yes	No	No	No	No	Scaled and comment
**Name of the UK website (n=14)**							
	Autumna	Yes	Yes	No	Yes	Yes (1,2,5)	Scaled
	Bestcarecompare	Yes	Yes	No	No	Yes (1-3,5)	Scaled and comment
	Bettercaring	Yes	Yes	No	Yes	No	No
	Bing	Yes	No	No	No	No	Scaled
	Carehome	Yes	Yes	No	Yes	Yes (1,2,5)	Scaled and comment
	Caresourcer	Yes	No	No	Yes	Yes (1,2,5)	Scaled and comment
	Google	Yes	No	No	No	No	Scaled and comment
	Hotfrog	Yes	No	No	No	No	No
	Housingcare	Yes	Yes	No	Yes	Yes	No
	Mostrecommendedcare	Yes	Yes	No	No	Yes (5)	Scaled and comment
	Trustedcare	Yes	No	No	No	Yes (2,5)	Scaled and comment

	Which	Yes	Yes	No	No	Yes (1,2,5)	No
	Yahoo	Yes	No	No	No	No	No
	Yell	Yes	No	No	No	No	Scaled and comment

Inspection results: 1=composite measure as overall rating; 2=composite measure as rating by area (eg, health inspections, staffing, and resident care); 3=detailed information from inspections by area; 4=reports for download; and 5=link to the authority website with detailed inspection result.
